# Supplementary material for: Comprehensive Annotation and Functional Exploration of MicroRNAs in Lettuce
Source: Front Plant Sci. 2021 Dec 24;12:781836. doi: 10.3389/fpls.2021.781836 (PMC8739914; doi:10.3389/fpls.2021.781836)
Supplement: Supplementary file 3 [file Data_Sheet_9.ZIP › GSM2230753.plot/Lsa-miRN533_Lsat_1_v5_gn_6_69800.1_2894_TPlot.pdf]

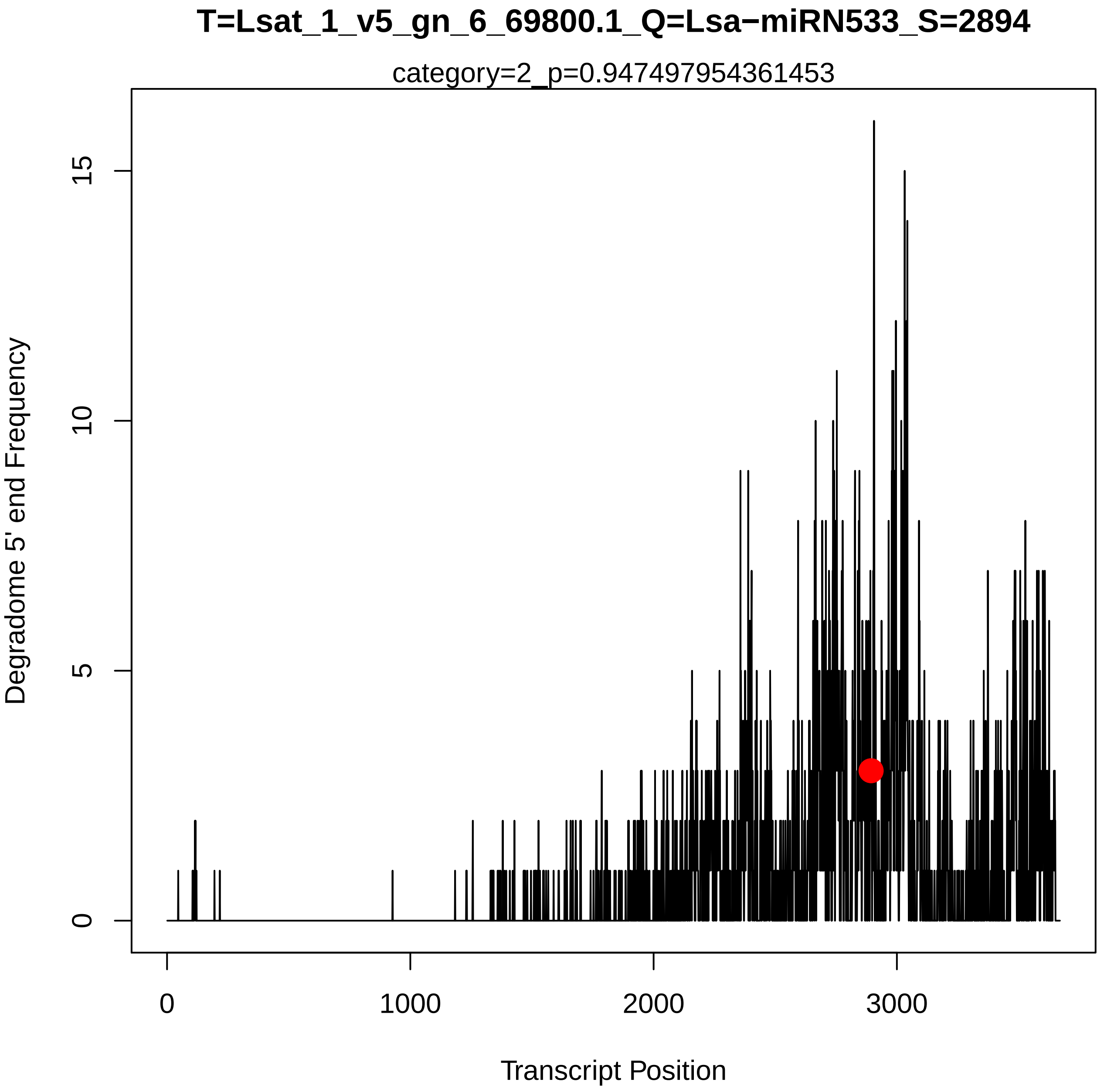

T=Lsat_1_v5_gn_6_69800.1_Q=Lsa−miRN533_S=2894
category=2_p=0.947497954361453
Degradome 5' end Frequency
0
5
10
15
0
1000
2000
3000
Transcript Position
